# Fibroblast growth factor 21 (FGF21) alleviates senescence, apoptosis, and extracellular matrix degradation in osteoarthritis via the SIRT1-mTOR signaling pathway

**DOI:** 10.1038/s41419-021-04157-x

**Published:** 2021-09-23

**Authors:** Hongwei Lu, Chao Jia, Dengying Wu, Haidong Jin, Zeng Lin, Jun Pan, Xiucui Li, Wei Wang

**Affiliations:** 1 Department of Orthopaedics, The Second Affiliated Hospital and Yuying Children’s Hospital of Wenzhou Medical University, Wenzhou, Zhejiang, 325027 Zhejiang Province China; 2 The Second School of Medicine, Wenzhou Medical University, Wenzhou, 325027 Zhejiang Province China; 3 Bone Research Institute, The Key Orthopaedic Laboratory of Zhejiang Province, Wenzhou, China; 4 Department of Neonatology, The Second Affiliated Hospital and Yuying Children’s Hospital of Wenzhou Medical University, Wenzhou, 325027 Zhejiang Province China

**Keywords:** Macroautophagy, Senescence

## Abstract

Osteoarthritis (OA) is a complex condition that involves both apoptosis and senescence and currently cannot be cured. Fibroblast growth factor 21 (FGF21), known for its role as a potent regulator of glucose and energy metabolism, protects from various diseases, possibly by mediating autophagy. In the present study, the role of FGF21 in the progression of OA was investigated in both in vitro and in vivo experiments. In vitro, the results revealed that FGF21 administration alleviated apoptosis, senescence, and extracellular matrix (ECM) catabolism of the chondrocytes induced by tert-butyl hydroperoxide (TBHP) by mediating autophagy flux. Furthermore, CQ, an autophagy flux inhibitor, could reverse the protective effect of FGF21. It was observed that the FGF21-induced autophagy flux enhancement was mediated by the nuclear translocation of TFEB, which occurs due to the activation of the SIRT1-mTOR signaling pathway. The in vivo experiments demonstrated that FGF21 treatment could reduce OA in the DMM model. Taken together, these findings suggest that FGF21 protects chondrocytes from apoptosis, senescence, and ECM catabolism via autophagy flux upregulation and also reduces OA development in vivo, demonstrating its potential as a therapeutic agent in OA.

## Introduction

Osteoarthritis (OA) is a degenerative disease of the joints that occurs in the elderly [1]. Until recently, no efficacious drugs and surgical procedures were available for OA therapy [2]. The incidence of osteoarthritis has increased sharply in the elderly population [3]. OA is reported to be a major cause of disability and socioeconomic loss worldwide [4]. Therefore, novel strategies to inhibit the progression of OA are of great clinical and scientific interest.

Articular cartilage is a complex tissue that is maintained by chondrocytes. As the only cell type in the articular cartilage, chondrocytes are responsible for producing the extracellular matrix (ECM) molecules [5]. Oxidative stress occurs due to the imbalance between the production of reactive oxygen species (ROS) and their elimination by the antioxidant defense system, which is high in the OA cartilage [6]. Excessive levels of ROS promote apoptosis, senescence, ECM catabolism, and ultimately leads to the degradation of the articular cartilage [7, 8]. Therefore, inhibiting oxidative stress of chondrocytes is proposed as a therapeutic target in OA.

Autophagy is a lysosome-dependent and highly-conserved macromolecular cycle occurring in the eukaryotic cells, which is also essential for the survival and maintenance of these cells [9]. It is characterized by the formation of double-layered vesicles (autophagosomes) around intracellular cargo for delivery to lysosomes and proteolytic degradation; the whole process is called autophagy flux [10]. Recent studies revealed that TFEB might regulate autophagy flux by inducing lysosomal biogenesis and promoting the formation of autophagosomes and their fusion with lysosomes [11, 12]. Interestingly, previous studies have shown that promoting the nuclear localization of TFEB in OA chondrocytes alleviated apoptosis and senescence through the autophagy lysosomal pathway (ALP) [13]. Thus, the promotion of the nuclear localization of TFEB and further activation of the autophagy flux could become the target of OA therapy.

In 2000, Nishimura et al. isolated the fibroblast growth factor 21 (FGF21) from mouse embryonic tissues [14]. Since then, FGF21 has attracted considerable attention as a therapeutic agent for the treatment of metabolic syndrome in humans [15–17]. In recent research, FGF21 has emerged as a longevity hormone [18]. FGF21 can stimulate autophagy in various tissues, including the brain [19], liver [20], random-pattern skin flaps [21], kidneys [22], heart [23], etc. Previous reports suggest that FGF21 has a potential therapeutic effect in neurodegeneration [24, 25]. However, as one of the degenerative diseases, no studies on the relationship between FGF21 and autophagy flux in osteoarthritis have been reported yet. Therefore, the present study was aimed to investigate the protective effects of FGF21 on the OA process, and it was hypothesized that these effects were exerted by the stimulation of autophagy flux.

In this study, tert-butyl hydroperoxide (TBHP) was used to induce oxidative stress as an exogenous ROS donor. We found that FGF21 might suppress apoptosis, senescence, and ECM catabolism induced by TBHP in chondrocytes. FGF21 might also promote autophagy flux and lysosome biogenesis, and these effects might be due to the activation of TFEB by FGF21 through the SIRT1/mTOR signaling pathway. Finally, the therapeutic potential of FGF21 was evaluated in the destabilization of the medial meniscus (DMM) mouse model.

## Results

### FGF21 reduced the apoptosis and senescence of the chondrocytes induced by TBHP

**Fig. 1:**
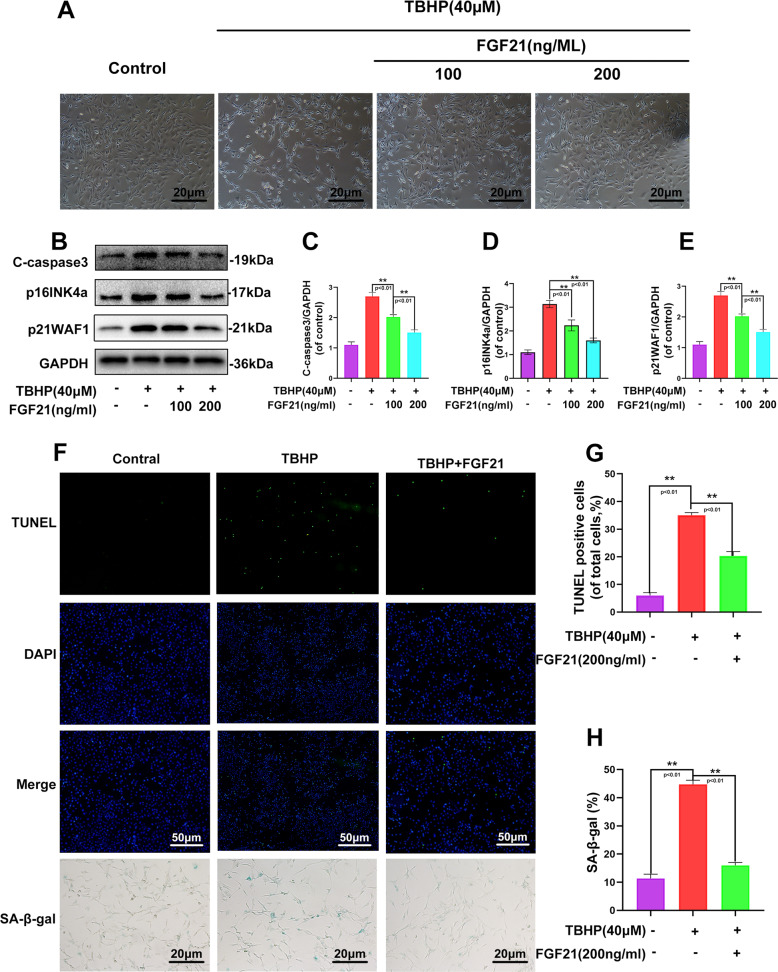
FGF21 treatment inhibited the apoptosis and senescence of TBHP-induced chondrocytes. **A** The chondrocytes were pretreated with FGF21 and subsequently with TBHP, and imaged using phase-contrast microscopy (scale bar: 20 μm). **B**–**E** Shown are protein contents of cleaved caspase3, p16INK4a, and p21WAF1 in the chondrocytes treated with TBHP and TBHP plus FGF21. **F**–**H** TUNEL assay and SA-β-gal staining assay were performed in the chondrocytes as treated above (scale bar: 50 μm). The data in the figures are the averages ± S.D. Significant differences between the treatment and control groups are indicated as **P < 0.01 and *P < 0.05, *n* = 3.

As depicted in Fig. 1A, after the application of TBHP, the chondrocytes shrunk in size, vacuole formation increased, and finally, the chondrocytes floated on the surface of the medium (Fig. 1A). In addition, the expression of the apoptosis-related protein cleaved-caspase3 and the classical senescence marker p16INK4a and p21WAF1 were analyzed, and it was observed that pretreatment with FGF21 inhibited the TBHP-induced increase in the protein content of cleaved-caspase3, p16INK4a, and p21WAF1 in a dose-dependent manner (Fig. 1B–E). The TUNEL assay demonstrated that FGF21 could reduce the incidence of apoptosis in the chondrocytes induced by TBHP (Fig. 1F, G). SA-β-gal staining assay further demonstrated that FGF21 could reduce the incidence of senescence in the chondrocytes induced by TBHP (Fig. 1F, H). These results indicated that FGF21 could protect the chondrocytes against the apoptosis and senescence induced by TBHP.

### FGF21 inhibited the catabolism of the ECM induced by TBHP in chondrocytes

**Fig. 2:**
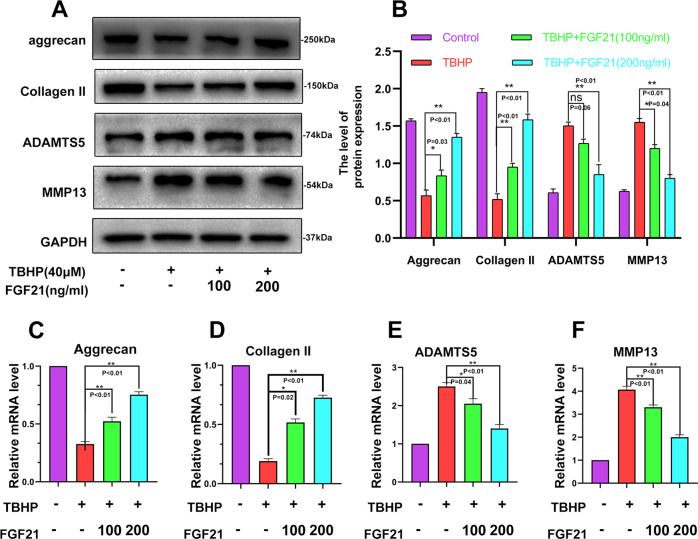
FGF21 treatment inhibited the TBHP-induced catabolism of the ECM. **A**, **B** Shown is the change in the levels of Aggrecan, Collagen II, ADAMTS5, and MMP13 in the chondrocytes, evaluated by the western blot analysis. **C**–**F** Shown is the mRNA expression of Aggrecan, Collagen II, ADAMTS5, and MMP13 in the chondrocytes treated with TBHP and TBHP plus FGF21, measured using real-time PCR. The data in the figures are the averages ± S.D. Significant differences between the treatment and control groups are indicated as **P < 0.01 and *P < 0.05, *n* = 3.

OA is closely associated with the catabolism of the ECM. Therefore, ECM-related proteins (Collagen II, Aggrecan, ADAMTS5, and MMP13) were analyzed by western blot. The results demonstrated that Collagen II and aggrecan were reduced in the TBHP group, while MMP-13 and ADAMTS-5 were enhanced in this group. FGF21 could suppress the expression of these mediators induced by TBHP in a dose-dependent manner (Fig. 2A, B). The real-time PCR results also demonstrated that TBHP treatment reduced the mRNA levels of collagen II and aggrecan. Furthermore, TBHP treatment increased the mRNA levels of MMP-13 and ADAMTS-5 (Fig. 2C–F). These results demonstrated that FGF21 inhibited the catabolism of the ECM induced in the chondrocytes by TBHP.

### FGF21 promoted autophagy flux in TBHP-treated chondrocytes

**Fig. 3:**
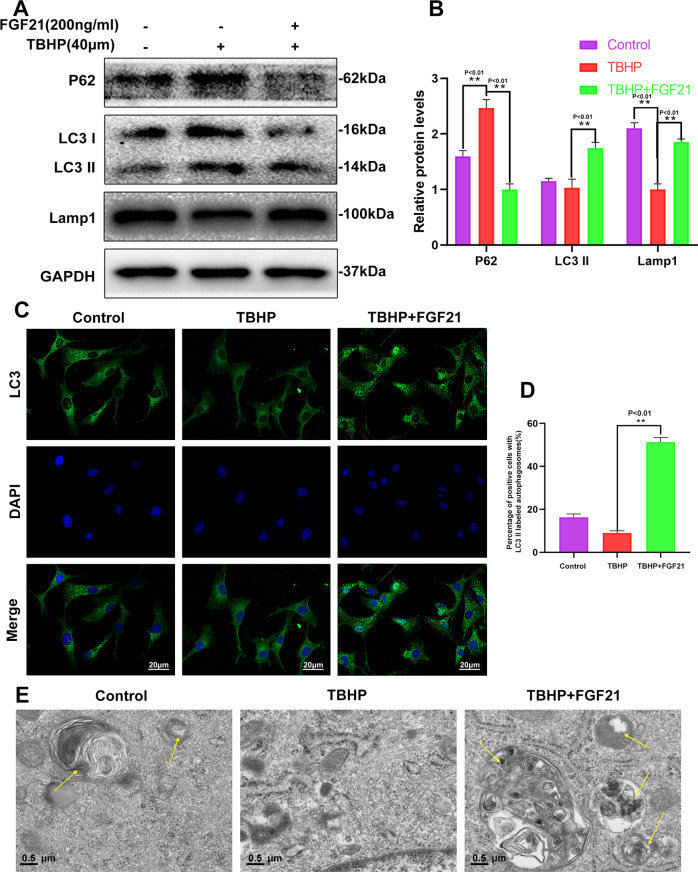
FGF21 treatment induced autophagy in chondrocytes. The chondrocytes were incubated with 100 or 200 ng/ml of TBHP only, or TBHP plus FGF21. **A**, **B** Shown is the content of Lamp1, LC3, and P62 in chondrocytes as treated above. **C** Shown are the TEM images of autophagic vesicles in chondrocytes (asterisk: autophagosome with double-membrane structure; triangle: autophagolysosome with single membrane structure) (scale bar: 0.5 µm). **D**, **E** The representative LC3-positive autophagic vesicles were detected by immunofluorescence staining (scale bar: 20 μm). The data in the figures are the averages ± S.D. Significant differences between the treatment and control groups are indicated as **P < 0.01 and *P < 0.05, *n* = 3.

Since FGF21 plays a role in ECM metabolism, apoptosis, and senescence, it was hypothesized that the effects of FGF21 could be, in part, due to the modulation of autophagy flux. LC3-II and SQSTM1/P62, which are regarded as the indicators of autophagy flux, and Lamp1, which is regarded as an indicator of lysosome biogenesis, were detected in the western blot assay. The results demonstrated that autophagy flux was blocked, and lysosome biogenesis was suppressed in the TBHP-treated chondrocytes. After the administration of FGF21 for 24 h, the expression of Lamp1 and LC3-II in the chondrocytes increased, while the level of SQSTM1/p62 decreased, compared to their levels after TBHP treatment only (Fig. 3A, B). In the immunofluorescence assay, it could also be seen that the expression level of LC3 II decreased after treatment with TBHP and increased after treatment with FGF21 (Fig. 3C, D). In addition, autophagosomes and autophagolysosomes were observed under a transmission electron microscope. There were more autophagy bodies and autophagy lysosomes in the cytoplasm of chondrocytes treated with FGF21 (Fig. 3E). These results collectively suggested that FGF21 promoted autophagy flux and lysosome biogenesis in chondrocytes.

### CQ reversed the protective effects of FGF21 against apoptosis and senescence induced by TBHP in chondrocytes

Chloroquine (CQ), a lysosomal cavity alkalizer, was found to block downstream autophagic flux in previous studies [26]. From the WB results, we found that CQ co-administration significantly decreased the expression of LC3 II/LC3 I, while it increased the expression of SQSTM1/p62 considerably (Fig. S1A, B). Therefore, CQ successfully decreased the autophagy flux induced by FGF21 in chondrocytes.

**Fig. 4:**
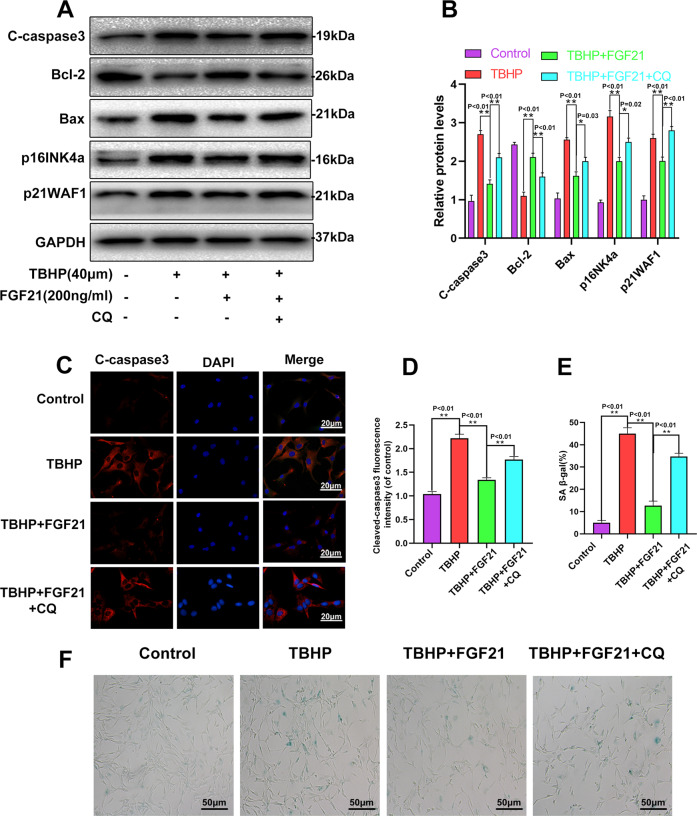
CQ reversed the effect of FGF21 on the apoptosis and senescence of chondrocytes under oxidative stress. **A**, **B** Shown is the protein expression of C-caspase3, Bax, Bcl-2, p16INK4a, and p21WAF1 in the chondrocytes treated as above. **C**, **D** The immunofluorescence analysis of C-caspase3 proteins in the chondrocytes as treated above (scale bar: 20 μm). **E**, **F** The SA-β-gal staining assay was performed in the chondrocytes as treated above (scale bar: 50 μm). The data in the figures are the averages ± S.D. Significant differences between the treatment and control groups are indicated as **P < 0.01 and *P < 0.05, *n* = 3.

Next, it was investigated whether autophagy flux was involved in the FGF21-induced protective effect against apoptosis and senescence in the chondrocytes. The results demonstrated that in the FGF21 + CQ group, the contents of C-caspase3, Bax, p16INK4a, and p21WAF1 increased significantly, while the level of Bcl-2 decreased, compared to their level in the FGF21 group (Fig. 4A, B). The immunofluorescence staining results demonstrated that compared to the FGF21-treated cells, CQ co-administration significantly reversed the expression of C-caspase3 (Fig. 4C, D). Finally, a significant increase in the SA-β-gal-positive senescent chondrocytes was observed following incubation with TBHP, and FGF21 significantly inhibited this increment. However, when autophagy flux was inhibited by CQ, the FGF21-induced anti-senescence effect was attenuated (Fig. 4E, F). These results indicated that FGF21 inhibited apoptosis and senescence induced in the chondrocytes by TBHP via autophagy flux.

### CQ reversed the protective effects of FGF21 on the ECM metabolism in the chondrocytes induced by TBHP

**Fig. 5:**
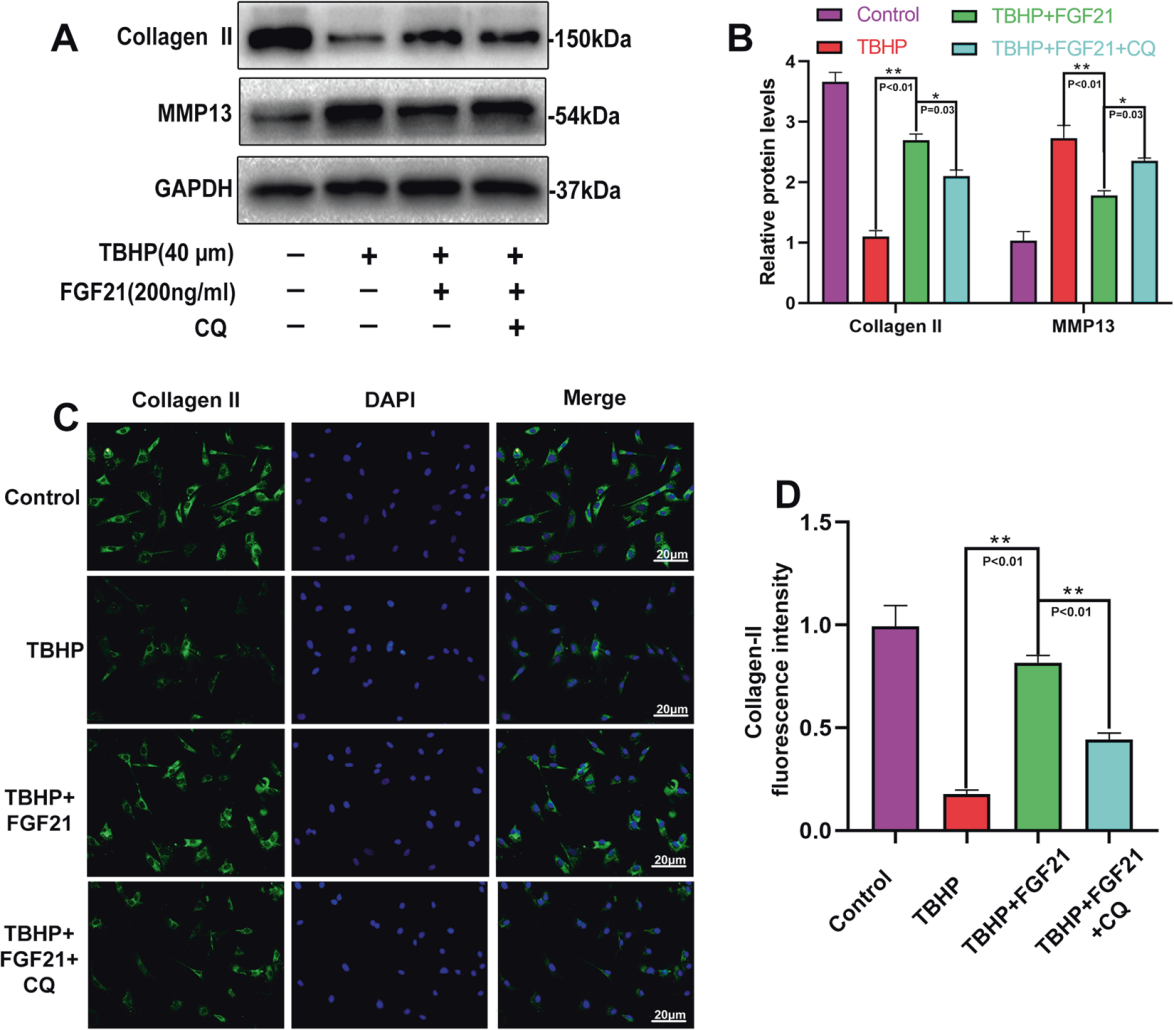
CQ reversed the effect of FGF21 on the ECM of chondrocytes under oxidative stress. **A**, **B** Shown are the changes in the levels of Collagen II and MMP13 in chondrocytes, evaluated using western blot analysis. **C**, **D** The representative Collagen II was detected using the immunofluorescence analysis combined with DAPI staining for the nuclei (scale bar of Collagen II: 20 μm; scale bar of MMP13: 20 μm). The data in the figures are the averages ± S.D. Significant differences between the treatment and control groups are indicated as **P < 0.01 and *P < 0.05, *n* = 3.

To further validate that autophagy flux was the main player in the beneficial effect of FGF21 in the osteoarthritis process, the effect of CQ co-administration on ECM metabolism was determined. It was observed that the levels of the protein related to the synthesis of the extracellular matrix (Collagen II) were reduced significantly, while the levels of the extracellular matrix decomposition related protein (MMP13) were increased in the FGF21 + CQ group compared to their levels in the FGF21 group, thus indicating that autophagy flux was involved in the protective effect of FGF21 on the catabolism of ECM in the chondrocytes (Fig. 5A, B). In addition, the immunofluorescence analysis results for collagen II protein expressions remained consistent with the WB results (Fig. 5C, D). Therefore, it was inferred that FGF21 prevented the catabolism of the ECM in the chondrocytes induced by TBHP via autophagy flux.

### FGF21 upregulated autophagy flux by enhancing TFEB activity

**Fig. 6:**
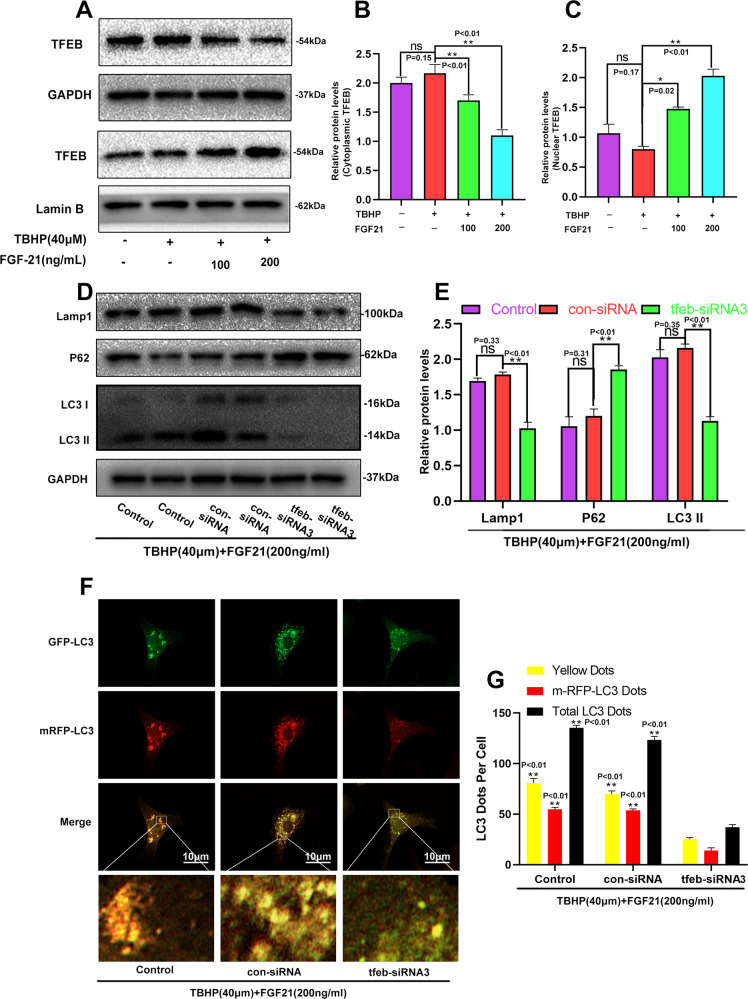
FGF21 upregulated autophagy flux by enhancing the TFEB activity. **A**–**C** Western blotting revealed the levels of cytoplasmic TFEB and nuclear TFEB in chondrocytes as treated above. **D**, **E** Western blotting revealed the levels of the autophagic proteins (p62 and LC3II) and Lysosomal protein (Lamp1) in chondrocytes as treated above. **F**, **G** The mRFP-GFP-LC3 assay was performed to detect the number of autophagosomes in chondrocytes (yellow signal represents autophagosomes; red signal represents autophagolysosomes; scale bar: 10 μm). The data in the figures are the averages ± S.D. Significant differences between the treatment and control groups are indicated as **P < 0.01 and *P < 0.05, *n* = 3.

Previous studies have demonstrated that FGF21 could activate TFEB to augment autophagy [21]. However, it was unknown whether this effect occurred in chondrocytes. The results of the western bolt analysis had also revealed that the intranuclear levels of TFEB were significantly increased by FGF21, while the levels of cytoplasmic TFEB were decreased (Fig. 6A–C). These results suggested that FGF21 might augment autophagy flux via TFEB activation. To further elucidate the role of TFEB in FGF21-induced augmentation of autophagy flux, chondrocytes pretreated with FGF21 and TBHP were transfected with three pairs of TFEB-siRNA to silence the TFEB activity. It was found that the expression of TFEB was successfully knocked down in the TFEB-siRNA3 pretreated chondrocytes (Fig. S2A–C). Next, western blotting and mRFP-GFP-LC3 fluorescence were performed to assess autophagy flux. In the TBHP + FGF21 treatment, silencing TFEB downregulated the protein contents of the Lamp1, and the ratio of LC3-II/LC3-I, while the expression of SQSTM1/p62 increased (Fig. 6D, E). In addition, compared to that in the control group and the con-siRNA group, the number of autophagosomes and autophagolysosomes was significantly reduced in the TFEB-siRNA3 group, indicating impaired autophagic flux with lysosomal dysfunction (Fig. 6F, G). Together, these results suggested that FGF21 promoted autophagic flux via TFEB in chondrocytes.

### The SIRT1-mTOR pathway was activated by FGF21 and was involved in the protective effect of FGF21 in the chondrocytes induced by TBHP

**Fig. 7:**
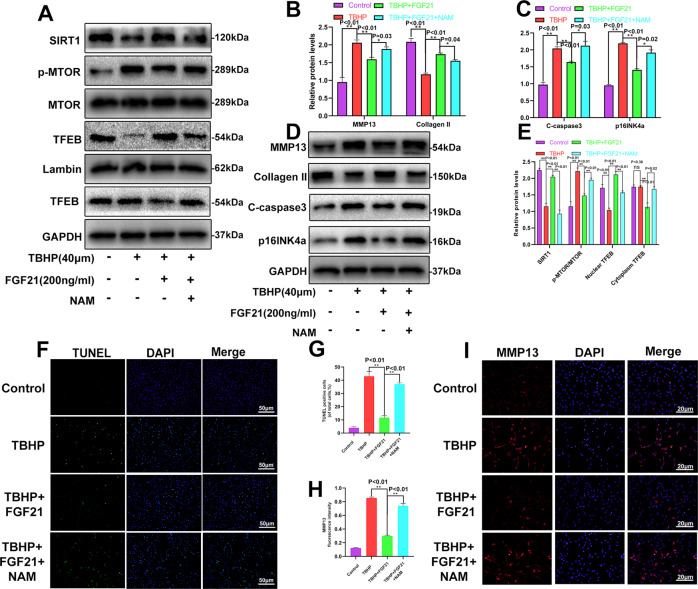
FGF21 activated the SIRT1-mTOR pathway in the chondrocytes induced by TBHP. **A**, **E** Western blotting revealed the levels of SIRT1, mTOR, p-mTOR, cytoplasmic TFEB, and nuclear TFEB in chondrocytes as treated above. **B**–**D** Shown is the protein expression of Collagen II, MMP13, C-caspase3, and p16INK4a in the chondrocytes as treated above. **F**, **G** TUNEL assay was performed in the chondrocytes as treated above (scale bar: 50 μm). **H**, **I** The representative MMP13 was detected using the immunofluorescence analysis combined with DAPI staining for the nuclei in the chondrocytes as treated above (scale bar for MMP13: 20 μm). Significant differences between the treatment and control groups are indicated as **P < 0.01 and *P < 0.05, *n* = 3.

According to previously published reports, the SIRT-mTOR pathway has a crucial role in TFEB modulation [27]. Therefore, we assumed that the SIRT1-mTOR pathway would be activated in the chondrocytes treated with FGF21. Our results showed that FGF21 increased the expression of SIRT1 and inhibited p-mTOR, indicating that FGF21 activated the SIRT1-mTOR pathway. To determine whether this FGF21-induced activation of TFEB was mediated by the SIRT1-mTOR signaling pathway in chondrocytes, nicotinamide (NAM), a SIRT1 blocker, was used for the inhibition of SIRT1 activation, and the results showed that FGF21 promoted the SIRT1-mTOR pathway-mediated TFEB nuclear translocation and these effects were reversed after NAM application (Fig. 7A, E). The results of western blotting also indicated that NAM significantly inhibited the FGF21-mediated protective effects on the ECM, apoptosis, and senescence (Fig. 7B–D). The TUNEL assay results and the immunofluorescence analysis further demonstrated the protective effect of FGF21 on apoptosis and ECM catabolism (Fig. 7F–I). Together, these results confirmed that FGF21 activated TFEB in the chondrocytes via the SIRT1-mTOR signaling pathway.

### FGF21 reduced OA development through the SIRT1-mTOR signaling pathway

**Fig. 8:**
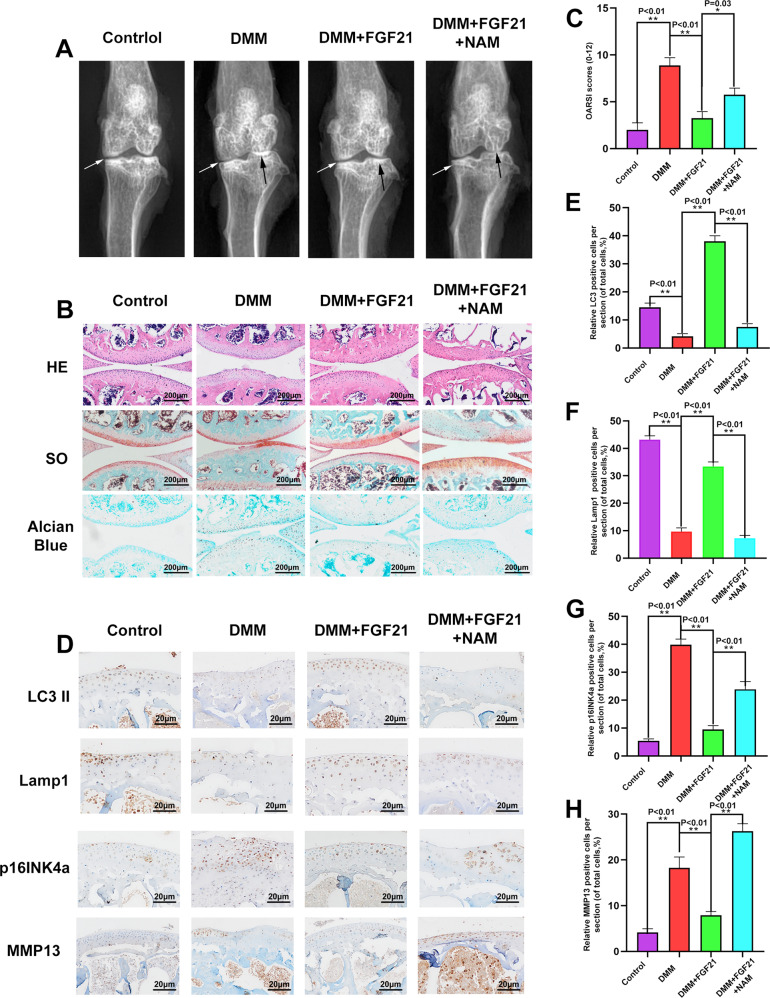
FGF21 inhibited OA progression in the DMM mouse model via the SIRT1-mTOR signaling pathway. **A** Shown are the digital X-ray images of the mouse knee joints from different groups. **B** Shown are the representative H&E, S-O, and Alcian Blue staining of cartilage in each group, 8 weeks after the surgery (scale bar: 200 μm). **C** Shown are the OARIS scores for the cartilages in each group. **D**–**H** Shown are the expressions of LC3, Lamp1, p16INK4a, and MMP13, evaluated using immunohistochemistry staining (scale bar: 20 μm). Significant differences between the treatment and control groups are indicated as **P < 0.01 and *P < 0.05, *n* = 6.

Based on the results of the in vitro experiment, we further studied the role of FGF21 in vivo, for which the DMM mouse model was used. The X-ray images obtained eight weeks after surgery revealed that the DMM group had a wider joint space compared to the DMM + FGF21 group. However, the DMM + FGF21 + NAM group had a narrower joint space compared to the DMM + FGF21 group (Fig. 8A). The protective effect of FGF21 observed on the histomorphology of knee joints in vivo was further confirmed from the results of the H&E and safranin O Fast Green staining and Alcian Blue staining, 8 weeks after the surgery. Erosion and hypocellularity of the superficial articular cartilage, along with proteoglycan loss, were observed in the DMM group. In contrast, the DMM + FGF21 group presented a more complete cartilage surface and richer proteoglycan (Fig. 8B). Consistent with the staining results, the OARSI score declined in the FGF21-treated group and increased in the DMM group. However, the combined treatment with NAM and FGF21 was observed to significantly offset the protective effect of FGF21 only on the articular cartilage structure and the matrix (Fig. 8C).

Furthermore, by immunohistochemistry, we found that the FGF21 group had significantly higher LC3 and Lamp1 expression and lower MMP13 and p16INK4a expressions in the chondrocytes compared to that in the DMM group (Fig. 8D–H). After the immunofluorescence staining of joint tissues, it was found that the FGF21 group exhibited significantly higher levels of TFEB in the nucleus of the chondrocytes, compared to that in the nucleus of the chondrocytes in the DMM group (Fig. S3A, B). The results of the TUNEL assay showed that the percentage of apoptosis in the FGF21 group was less than that in the DMM group (Fig. S3C, D). However, the NAM and FGF21 co-treatment groups exhibited a greater offset than the effect of FGF21 on the results of the above-mentioned chondrocytes, which was consistent with the results of the X-ray examination and histological staining. To summarize, these results demonstrated the therapeutic effect of FGF21 in vivo.

## Discussion

Even though OA is one of the most common musculoskeletal diseases [28], there is no effective treatment available for it so far because of its complex pathology and side effects of the drugs [2]. Currently, non-steroidal anti-inflammatory drugs are used to relieve the symptoms of OA [29]. However, these drugs do not effectively prevent or decelerate the progress of OA. Therefore, it is imperative to develop further effective drugs to prevent and treat OA. The present study demonstrated that FGF21 might increase the levels of TFEB by activating the SIRT1-mTOR signaling pathway and subsequently promote the autophagy flux of chondrocytes, thereby reducing the TBHP-induced apoptosis and senescence. In addition, FGF21 effectively reduced the progression of DMM-induced OA in vivo.

FGF21, an important member of the FGF family and an important cytokine, is demonstrated to respond to nutrition and exercise and has also been reported to play an important role in improving glucose and lipid metabolism [20, 30, 31]. Previous studies have also demonstrated that as a potent longevity factor, FGF21 treatment can alleviate many age-related diseases, and have a role in the inhibition of senescence and inflammation in several diseases [25, 32, 33]. In addition, Li et al. observed OA patients had significantly higher serum and synovial fluid FGF21 concentration compared to that in the controls [34]. Paré et al. found a potential beneficial effect of FGF-21 against OA [35]. Given the potential therapeutic effect of FGF21, we hypothesized that FGF21 might reduce the progression of osteoarthritis, which was verified in the present study.

In this study, we used TBHP as a ROS donor to stimulate apoptosis and stress-induced premature senescence in chondrocytes in the in vitro study [36, 37]. Apoptosis is well-recognized as a key regulatory factor in the occurrence and development of OA [38, 39]. It was observed that FGF21 pretreatment could significantly reduce the levels of Bax and C-caspase3 while increasing the Bcl-2 levels. In addition, the TUNEL staining results revealed that the FGF21-treated and TBHP-induced DNA damage in the chondrocytes decreased, which could be a consequence of the inhibition of apoptosis. Senescence is considered to be another key regulator in the process of OA [40, 41]. Cyclin-dependent kinase inhibitors p16INK4a and p21WAF1 are the typical regulators of senescence [42, 43]. The results of the present study revealed that FGF21 could significantly reduce the expression of p16INK4a and p21WAF1. The SA-β-GAL staining results of our study further revealed that the presence of FGF21 significantly inhibited oxidative stress-induced senescence. Furthermore, we demonstrated that FGF21 treatment markedly inhibited the TBHP-induced ECM catabolism, thereby assisting in maintaining the balance of the articular cartilage environment. Our study found that FGF21 could alleviate apoptosis, senescence, and ECM catabolism by promoting the nuclear translocation of TFEB in chondrocytes.

In recent years, strong evidence has emerged demonstrating that moderate autophagy flux provides a potential therapeutic target for OA, as it might help to alleviate apoptosis and senescence [13, 44]. Tang et al. observed in human OA cartilage and DMM mouse OA model that the expression of p62 increased significantly, thus suggesting that catabolism of OA chondrocytes by autophagy was blocked [44]. Moreover, from the studies by Kim et al. and Ansari et al., it was found that compared to that in the normal chondrocytes, human and mouse OA chondrocytes had greater lysosomal accumulation and greater lysosomal dysfunction [45, 46]. The destruction of lysosomes led to chondrocyte apoptosis and autophagy inhibition [47]. These results indicated that promoting the formation of autophagosomes and improving the function of lysosomes might restore autophagic flux, which might, in turn, protect chondrocytes against cellular damage.

TFEB is a basic spiral-cyclic-leucine zipper (bHLH-Zip) transcription factor, and its activity and subcellular localization rely on its phosphorylation level [48]. Under normal physiological conditions, TFEB is phosphorylated mainly by the mechanical target molecules of the rapamycin complex 1 (mTORC1), following which TFEB binds to chaperone 14–3–3, thus being isolated in the cytoplasm [49]. However, under a stressful environment, such as hunger, oxidative stress, toxicity, etc., the activity of mTORC1 is inhibited. Subsequently, the dephosphorylated TFEB is transferred to the nucleus and promotes the expression of various autophagy-related genes and lysosomal genes, which in turn promote autophagy and lysosomal biogenesis [48, 50]. In the present study, FGF21 was demonstrated to promote the formation of autophagosomes and restore lysosomal function by promoting the nuclear translocation of TFEB.

Furthermore, the mechanism by which FGF21 regulates the level of TFEB was also studied. It has been reported that SIRT1-mediated downregulation of mTOR signal enhances nuclear transcription of TFEB and increases autophagosomes [27, 51]. Interestingly, we found that the TBHP-induced chondrocytes pretreated with FGF21 activated the SIRT1-mTOR signaling pathway, increased the nuclear translocation of TFEB, inhibited apoptosis and senescence of the chondrocytes, and decelerated the progress of OA in vivo. Moreover, nicotinamide (NAM), a SIRT1 blocker, could reverse these effects of FGF21. Therefore, it was inferred that FGF21 could promote the nuclear translocation of TFEB through the activation of the SIRT1-mTOR signaling pathway.

The surgical model of destabilization of the medial meniscus (DMM) has become an essential model for studying apoptosis and senescence in posttraumatic osteoarthritis (OA) [13, 52]. In the in vivo experimental DMM model, several in vivo evaluation methods such as X-ray, H&E staining, SO staining, Alcian Blue staining, immunochemistry, tissue TUNEL assay, and tissue fluorescence were used to determine the effect of FGF21 on the progression of OA in mice. The results were almost consistent with in vitro experiments, which indicated that FGF21 might inhibit the occurrence and development of OA by activating the SIRT1-mTOR signaling pathway, thereby promoting nuclear translocation of TFEB (Fig. S4).

Although our study demonstrated that FGF21 reduces senescence, apoptosis, and ECM catabolism in TBHP-treated chondrocytes via the SIRT1-mTOR signaling pathway, there are still several limitations of our study. First, although we found that FGF21 had a positive effect on the progression of osteoarthritis, the chondrocytes isolated from the articular cartilage showed the particularity of differentiation [53]. Chondrocytes treated with TBHP only partially reflected the pathological condition of osteoarthritis. Second, the mechanism of the effects of FGF21 on chondrocytes is still unclear. FGF21-mediated inhibition of TBHP-induced apoptosis and senescence might also result from antioxidative effects of the growth factor. A DPPH assay would clarify this issue. Therefore, it is necessary to further study the effect of FGF21 on chondrocytes in the future.

In summary, our results not only suggested that FGF21 could serve as an effective treatment tool for inhibiting the progression of OA, but it could also provide insights into the mechanism of action of FGF21 in chondrocytes. The present study highlights the potential of FGF21 in clinical transformation, particularly in the treatment of OA in the future.

## Materials and methods

### Antibodies and reagents

FGF21 (P6101) was obtained from Beyotime Biotechnology (Jiangsu, China), chloroquine (CQ) was obtained from Sigma-Aldrich Chemical Company (Milwaukee, WI, USA), and nicotinamide (NAM) was purchased from MedChemExpress (NJ, USA). The primary antibodies directed against Collagen II, MMP-13, p16INK4a, p21WAF1, Lamin B1, and GAPDH were purchased from Abcam (Cambridge, MA, USA), while the primary antibodies directed against Bax, Bcl-2, SQSTM1/p62, and LC3 were obtained from ProteinTech (Wuhan, China). The antibodies against cleaved caspase3 (C-caspase 3), TFEB, mTOR, Phospho-mTOR (p-mTOR), and SIRT1 were procured from Cell Signaling Technologies (Danvers, MA, USA).

### Isolation and primary culture of chondrocytes

Six immature C57BL/6 mice (3 males and 3 females, 10-day-old) were sacrificed, the articular cartilage was separated under sterile conditions and carefully collected. Briefly, the cartilages of the knee joints of the mice were cut into pieces (1 mm^3^) and then enzymatically digested in 2 mg/mL of 0.1% collagenase II at 37 °C for 4 h. The digested cartilage tissue pieces were suspended in DMEM/F12 (Gibco, Invitrogen, Grand Island, NY) medium supplemented with 10% fetal bovine serum (FBS; HyClone, Thermo Scientific, Logan, UT, USA) and 1% penicillin/streptomycin (Gibco, Invitrogen, Grand Island, NY), and seeded into tissue culture flasks. The chondrocytes were grown in an incubator maintained at 37 °C under 5% CO_2_ conditions. After 24 h of incubation, the culture medium was changed, and the second- or third-generation cells were retrieved for the subsequent experiments.

### Animal model

A total of 60 ten-week-old C57BL/6 male wild-type (WT) mice were procured from the Animal Center of the Chinese Academy of Sciences Shanghai, China. The mice were randomly divided into four groups (*n* = 15 in each) as the Control group (sham-operated), the DMM group (OA), the DMM + FGF-21 group (OA treated with FGF-21), and the OA + FGF-21 + NAM (OA treated with FGF-21 and NAM) group. After anesthetization with 2% (w/v) pentobarbital (40 mg/kg), as mentioned earlier [52], the mouse model of osteoarthritis was developed by surgical destabilization of the medial meniscus (DMM). Eight weeks after the DMM surgery, the mice in each group were sacrificed, and their joints were subjected to histological evaluation.

### Western blot assay

The total protein in the chondrocytes was extracted using the RIPA lysis buffer with 1 mM PMSF (phenylmethanesulfonyl fluoride). The protein concentration was determined using the BCA protein assay kit (Beyotime). The protein (40 ng) was separated on sodium dodecyl sulfate-polyacrylamide gel electrophoresis (SDS-PAGE) gels and transferred to a polyvinylidene difluoride (PVDF) membrane. After blocking the membrane by incubation with 5% non-fat milk for 2 h, the membrane was incubated overnight at 4 °C with the following primary antibodies: aggrecan (1:1000), collagen II (1:1000), ADAMTS-5 (1:1000), MMP-13 (1:1000), p16INK4a (1:1000), p21WAF1 (1:1000), Lamin B1 (1:1000), GAPDH (1:5000), Bax (1:1000), Bcl-2 (1:1000), P62 (1:1000), Lc3 (1:1000), C-caspase3 (1:1000), TFEB (1:1000), mTOR (1:1000), Phospho-mTOR (p-mTOR) (1:1000), and SIRT1 (1:1000). Next, the bands were incubated with the respective secondary antibodies for 2 h at room temperature, followed by three washes with TBST, and then visualized using an electrochemiluminescence reagent (Invitrogen). Finally, the intensity of these blots was determined using Image Lab 3.0 software (Bio-Rad).

### TUNEL staining

The apoptosis of chondrocytes was evaluated using an in-situ Cell Death Detection Kit (Roche, South San Francisco, CA). The chondrocytes were fixed in 4% paraformaldehyde for ~1 h and then incubated for 10 min each with 3% H_2_O_2_ and 0.2% Triton X-100. After three washes with PBS, the cells were stained using the TUNEL staining solution and DAPI. Finally, the apoptosis of the chondrocytes was observed under an Olympus fluorescence microscope (Olympus Inc., Tokyo, Japan).

### Transmission electron microscopy

The chondrocytes were fixed overnight in 2.5% glutaraldehyde, post-fixed in 2% osmium tetroxide for 1 h, and finally stained with 2% uranyl acetate for 1 h. After dehydration in a series of acetone solutions, the samples were embedded in Araldite and excised into semi-thin sections, which were stained with toluidine blue to locate the cell position and were finally observed under a transmission electron microscope (Hitachi, Tokyo, Japan). In each section, 30 cells were selected randomly, and their image was captured.

### Transduction with mRFP-GFP-LC3 and analysis

Before co-culturing, the chondrocytes were cultured in a lower compartment, and (GeneChem, Shanghai, China) when 50–70% confluence was reached, the cells were transduced with mRFP-GFP-LC3, according to the instructions of the manufacturer. After the incubation, the autophagosomes in the chondrocytes were observed under a confocal microscope (Leica TCS SP8, Germany), and the total number of puncta (1 mm) per cell was counted.

### Immunofluorescence

Chondrocytes were seeded in six-well plates on glass coverslips, followed by washing with PBS, fixing in 4% paraformaldehyde, and permeation in 0.1% Triton X-100 for 15 min. After blocking with 5% bovine serum albumin for 30 min, the chondrocytes were incubated overnight at 4 °C with the primary antibody against LC3 (1:200), cleaved-caspase3 (1:400), MMP13 (1:100), or collagen II (1:100). The following day, the cells were washed and incubated with Alexa Fluor 488or Alexa Fluor 594 for 1 h at room temperature for labeling. The slides were observed under a fluorescence microscope (Olympus Inc., Tokyo, Japan), and the fluorescence intensity was measured using the Image J software 2.1 (Bethesda, MD, USA).

### X-ray imaging method

Eight weeks after the surgery, with or without treatment, the mice were subjected to X-ray examination using a digital X-ray machine (Kubtec Model PERT.8; KUB Technologies Inc.) to evaluate the articular surface, osteophyte formation, and calcification changes on the cartilage surface. Appropriate images were obtained at 50 kV and 160 µA.

### Statistical analysis

The results were expressed as mean ± S.D. The data were analyzed using the SPSS statistical software program 18.0. The differences among the groups were determined by performing a one-way analysis of variance (ANOVA) or *t*-test. Statistical differences among and/or between groups were considered at *P* < 0.05. All the experiments were performed independently three times and were consistently repeatable.

## Supplementary information

supplementary material (methods)

Supplementary materials (figure legends)

Fig. S1.

Fig. S2.

Fig. S3.

Fig. S4.

## Data Availability

The datasets used and/or analyzed during the current study are available from the corresponding author on reasonable request.
