# Supplementary material for: Fibroblast growth factor 21 (FGF21) alleviates senescence, apoptosis, and extracellular matrix degradation in osteoarthritis via the SIRT1-mTOR signaling pathway
Source: Cell Death Dis. 2021 Sep 23;12(10):865. doi: 10.1038/s41419-021-04157-x (PMC8460788; doi:10.1038/s41419-021-04157-x)
Supplement: Supplementary file 1 — supplementary material (methods) [file 41419_2021_4157_MOESM1_ESM.docx]

**Antibodies and reagents**

Anti-TFEB, Goat anti-rabbit, and anti-mouse IgG-HRP were obtained from Bioworld (OH, USA). Horseradish peroxidase-labeled secondary antibodies, Alexa Fluor® 488-labeled goat anti-mouse IgG (H + L) secondary antibody, and Alexa Fluor® 594-labeled goat anti-rabbit IgG (H + L) secondary antibody were purchased from Abcam. The 4’, 6-diamidino-2-phenylindole (DAPI) was obtained from Beyotime (Shanghai, China), and the mRFP-GFP-LC3 was obtained from Shanghai Genechem Co., Ltd. The cell culture reagents were purchased from Gibco (Grand Island, NY, USA).

**Experimental design**

In the in vitro experiments, the chondrocytes treated with different concentrations of FGF-21 (100, 200 ng/mL) for 24 h were subjected to TBHP (40 µM) administration to determine the protective effect of FGF-21 in the chondrocytes. Furthermore, TBHP-induced chondrocytes were treated with FGF21 to determine the role of FGF21 in autophagy flux activation. The si-TFEB and CQ (50 µM) treatments were performed for 24 h before the administration of FGF21 to confirm the protective effect of the autophagy flux activation by FGF21. To study the role of the SIRT1-mTOR pathway on the effect of FGF21, the chondrocytes were treated with FGF21 only, or with FGF21 along with a pretreatment using NAM (10 MM) for 24 h.

In the in vivo experiments, the mice were divided randomly into four groups (n = 15 per group) as the control group treated with phosphate buffer solution (PBS), the DMM group treated with phosphate buffer solution (PBS), the DMM + FGF21 group treated with FGF21 (100 µg/kg/day), the DMM + FGF21 + NAM group treated with FGF21 (100 µg/kg/day), and the NAM (1 mg/kg/d) group. PBS or FGF21 or FGF21+ NAM (150 μl) was intraperitoneally injected once daily for eight weeks. All mice were sacrificed after eight weeks post-puncture, and their joint samples were collected for histological analysis.

**Real-time PCR**

Total RNA was extracted from human chondrocytes using the Trizol reagent (Invitrogen), placed in a six-well plate, and reverse-transcribed and amplified by performing quantitative real-time polymerase chain reaction (qRT-PCR) according to standard protocols. The results were assessed using the 2−ΔΔCt method. The primer sequences used for the amplification of collagen II, Aggrecan, ADAMTS-5, MMP-13, and GAPDH were: collagen II (F) 5′-CTCAAGTCGCTGAACAACCA-3′, (R) 5′-GTCTCCGCTCTTCCACTCTG-3′; aggrecan (F) 5′-AAGTGCTATGCTGGCTGGTT-3′, (R) 5′-GGTCTGGTTGGGGTAGAGGT-5′; MMP13 (F) 5′-CCAGAACTTCCCAACCAT-3′, (R) 5′-ACCCTCCATAATGTCATACC-3′; ADTAMTS4 (F) 5′-GGGAATAAGTACTGGGCTGTTCAG-3′, (R) 5′-CCTCAGAAAGAGCAGCATCGATATG-3′; and GAPDH (F) 5′-TCTCCTCTGACTTCAACAGCGAC-3′, (R) 5′-CCCTGTTGCTGTAGCCAAATTC-3′.

**Histological assessment and immunohistochemistry**

Briefly, the knee joint samples from each group were fixed in 4% paraformaldehyde for 48 h at 4°C, followed by decalcification in 10% EDTA solution for four weeks. The 5 µm-thick sections obtained from across the entire joint were stained with Safranin-O–Fast green stain (S-O) and H&E to evaluate the cellularity and morphology of the cartilage and subchondral bone. The Osteoarthritis Research Society International (OARSI) scoring system was used for the evaluation of the medial femoral condyle and the medial tibial plateau, as described previously^53^. Fifteen mice from each group were selected for histomorphometric scoring. The mice cartilage sections (5 µm thick) embedded in paraffin were deparaffinized using xylene, then rehydrated by incubation with a graded ethanol series and endogenous peroxidase, and finally treated with 3% (v/v) hydrogen peroxide for 10 min. Antigen retrieval from the sections was achieved by incubation with 0.4% pepsin (Sangon Biotech, Shanghai, China) in 5 mM HCl at 37°C for 20 min. The sections were incubated with 5% bovine serum albumin for 30 min at room temperature, then with primary antibody ov

ernight at 4°C, and finally with HRP-conjugated secondary antibodies. The rate of positive cells per section was quantitated by the observers who were blinded to the experimental groups. The captured images were analyzed using the Image-Pro Plus software, version 6.0 (Media Cybernetics, Rockville, MD, USA).

**SA-β-gal staining**

The level of senescence was measured using the SA-β-gal staining kit (Beyotime, Shanghai, China) according to the manufacturer’s instructions. The aging cells with high SA-β-gal activity were stained blue in the assay. The images were observed under an optical microscope (Olympus Inc., Tokyo, Japan), and the percentage of SA-β-gal-positive senescent chondrocytes was measured using the Image J software 2.1 (Bethesda, MD, USA).

**TFEB siRNA cell transfection**

The siRNA for the mouse TFEB gene was purchased from Santa Cruz Biotechnology (Dallas, TX, USA). According to the manufacturer’s instructions, the chondrocytes were seeded in a six-well plate and cultured for 24 h to reach 60%–70% confluency. Subsequently, the cells were transfected with 50 nM negative control or TFEB siRNA duplexes using Lipofectamine 2000 siRNA transfection reagent (Thermo Fisher, UT, USA). After transfection, the chondrocytes were further treated and harvested for the Western blot experiments.
