# Supplementary material for: Fibroblast growth factor 21 (FGF21) alleviates senescence, apoptosis, and extracellular matrix degradation in osteoarthritis via the SIRT1-mTOR signaling pathway
Source: Cell Death Dis. 2021 Sep 23;12(10):865. doi: 10.1038/s41419-021-04157-x (PMC8460788; doi:10.1038/s41419-021-04157-x)
Supplement: Supplementary file 2 — Supplementary materials (figure legends) [file 41419_2021_4157_MOESM2_ESM.docx]

**Fig. S1. CQ reversed the effect of FGF21 on the autophagy flux of chondrocytes under oxidative stress.**

**(A, B)** The protein expression of LC3 II, and p62 in the chondrocytes treated as described above. Significant differences between the treatment and control groups are indicated as **P ＜ 0.01 and *P ＜ 0.05, n = 3.

**Fig. S2. The chondrocytes were transfected with TFEB-siRNA to silence the TFEB activity.**

**(A, B, C)** Western blotting reveals the levels of cytoplasmic TFEB and nuclear TFEB in the chondrocytes treated as described above. Significant differences between the treatment and control groups are indicated as **P ＜ 0.01 and *P ＜ 0.05, n = 3.

**Fig. S3. FGF21 ameliorates OA development through the SIRT1-mTOR signaling pathway.**

**(A, B)** Tissue immunofluorescence analysis results reveal the expression of TFEB translocation into the nucleus in in the chondrocytes treated as described above. (scale bar: 50 μm). **(C, D)** The TUNEL assay was performed with mouse joint tissue samples as treated above (scale bar: 50 μm). Significant differences between the treatment and control groups are indicated as **P ＜ 0.01 and *P ＜ 0.05, n = 3.

**Fig. S4. Schematic illustration of effect of FGF21 in osteoarthritis development.**
