# Supplementary figures and images for: Fibroblast growth factor 21 (FGF21) alleviates senescence, apoptosis, and extracellular matrix degradation in osteoarthritis via the SIRT1-mTOR signaling pathway
Source: Cell Death Dis. 2021 Sep 23;12(10):865. doi: 10.1038/s41419-021-04157-x (PMC8460788; doi:10.1038/s41419-021-04157-x)

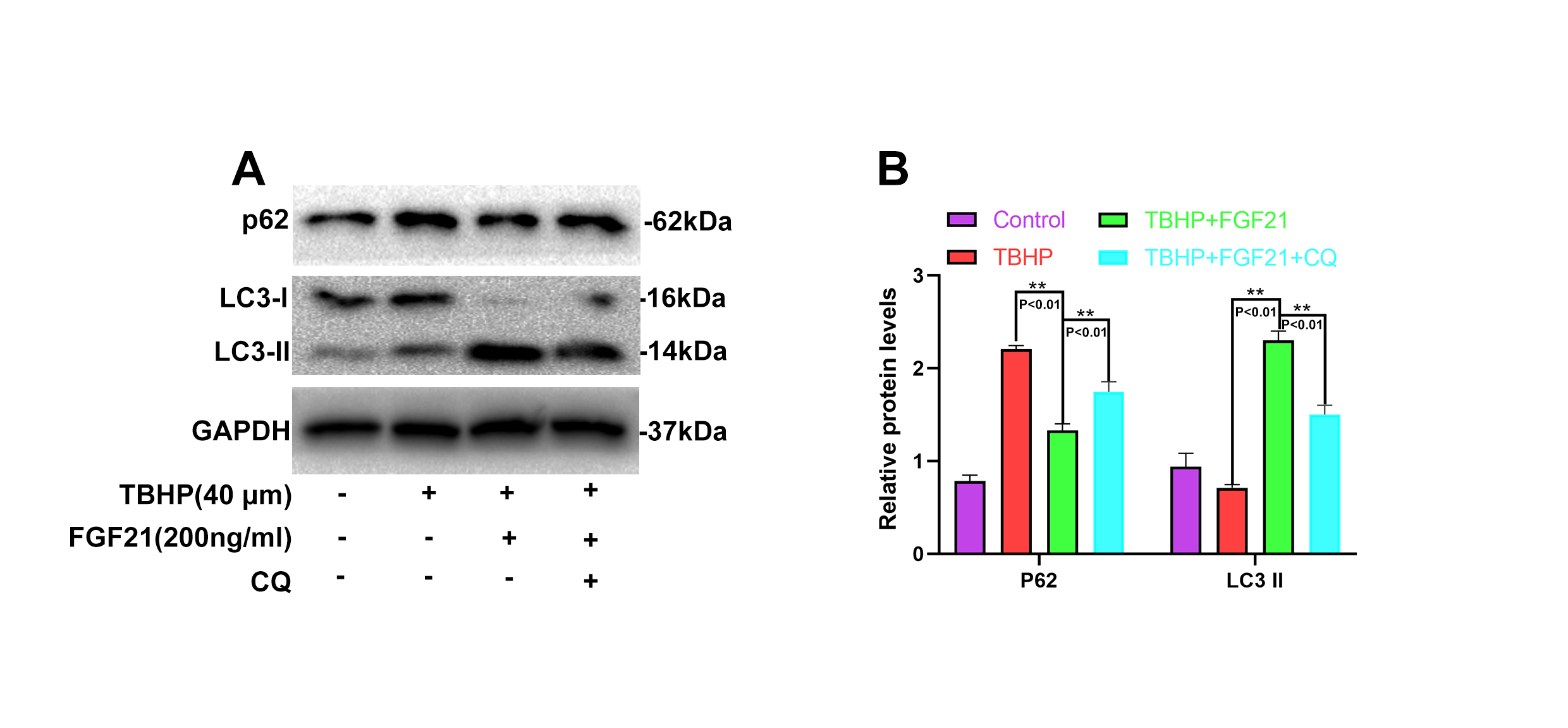

Supplement: Supplementary file 3 — Fig. S1. [file 41419_2021_4157_MOESM3_ESM.tif]

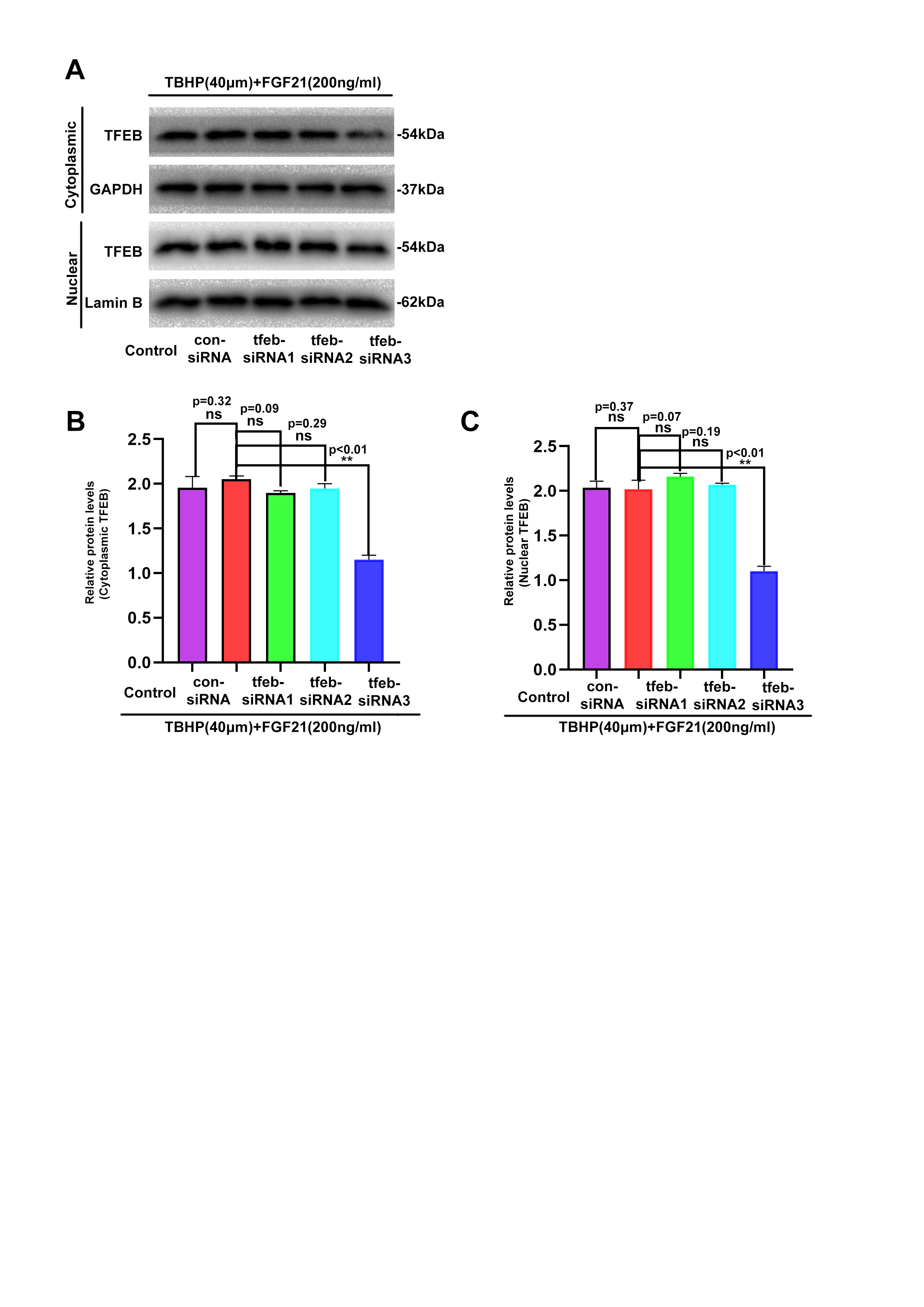

Supplement: Supplementary file 4 — Fig. S2. [file 41419_2021_4157_MOESM4_ESM.tif]

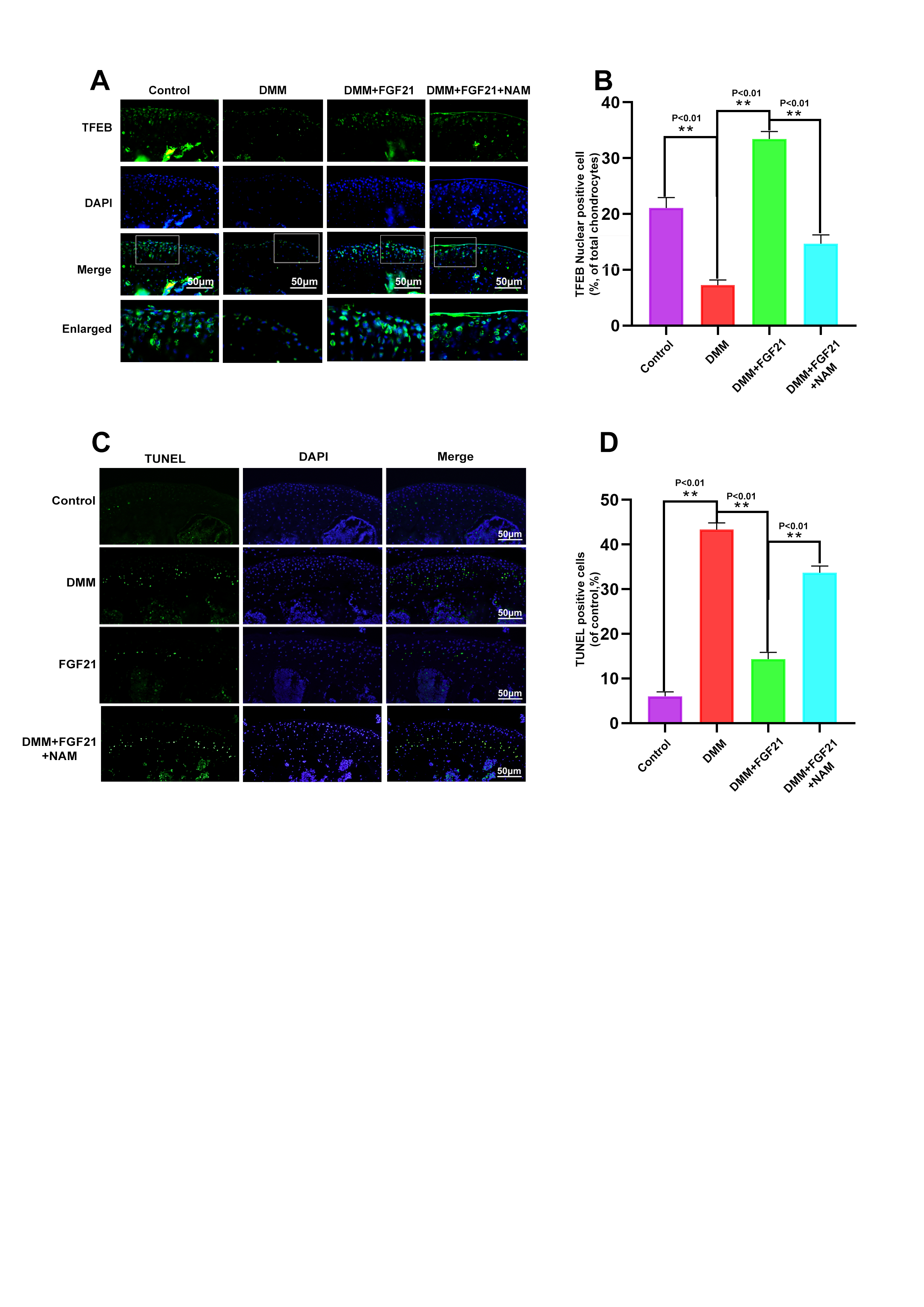

Supplement: Supplementary file 5 — Fig. S3. [file 41419_2021_4157_MOESM5_ESM.tif]

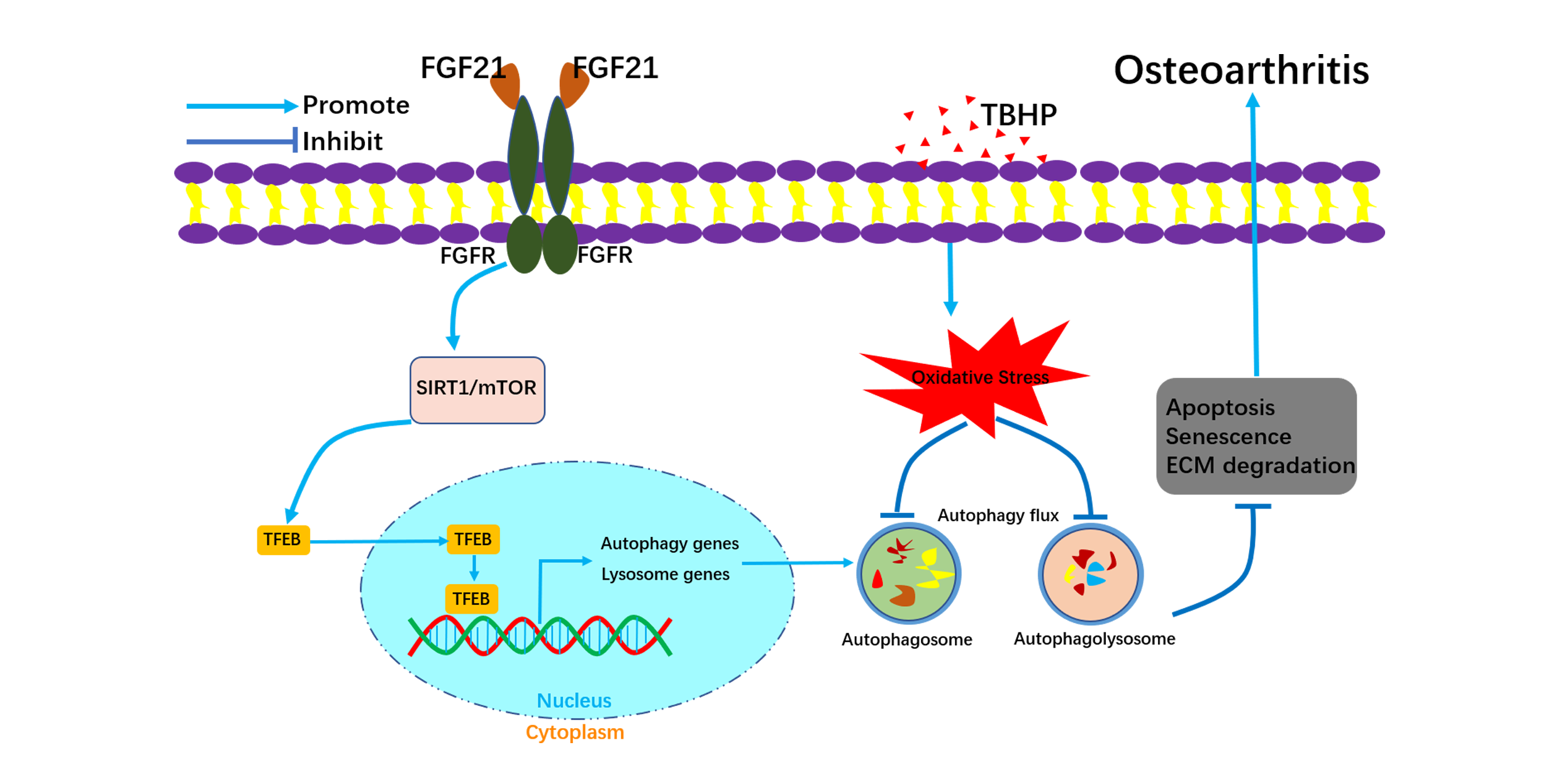

Supplement: Supplementary file 6 — Fig. S4. [file 41419_2021_4157_MOESM6_ESM.tif]
